# Pterostilbene Enhances Endurance Capacity via Promoting Skeletal Muscle Adaptations to Exercise Training in Rats

**DOI:** 10.3390/molecules25010186

**Published:** 2020-01-02

**Authors:** Jiawei Zheng, Wujian Liu, Xiaohui Zhu, Li Ran, Hedong Lang, Long Yi, Mantian Mi, Jundong Zhu

**Affiliations:** Research Center for Nutrition and Food safety, Chongqing Key Laboratory of Nutrition and Food Safety, Institute of Military Preventive Medicine, Third Military Medical University, Chongqing 400038, China; zhengjiawei@tmmu.edu.cn (J.Z.); liuyiyi0118@163.com (W.L.); zhu_xh@yeah.net (X.Z.); ran_li1983@163.com (L.R.); lang0401@163.com (H.L.); longgyin8341@hotmail.com (L.Y.)

**Keywords:** endurance capacity, muscle fiber type, angiogenesis, pterostilbene

## Abstract

It has been demonstrated that skeletal muscle adaptions, including muscle fibers transition, angiogenesis, and mitochondrial biogenesis are involved in the regular exercise-induced improvement of endurance capacity and metabolic status. Herein, we investigated the effects of pterostilbene (PST) supplementation on skeletal muscle adaptations to exercise training in rats. Six-week-old male Sprague Dawley rats were randomly divided into a sedentary control group (Sed), an exercise training group (Ex), and exercise training combined with 50 mg/kg PST (Ex + PST) treatment group. After 4 weeks of intervention, an exhaustive running test was performed, and muscle fiber type transformation, angiogenesis, and mitochondrial content in the soleus muscle were measured. Additionally, the effects of PST on muscle fiber transformation, paracrine regulation of angiogenesis, and mitochondrial function were tested in vitro using C2C12 myotubes. In vivo study showed that exercise training resulted in significant increases in time-to-exhaustion, the proportion of slow-twitch fibers, muscular angiogenesis, and mitochondrial biogenesis in rats, and these effects induced by exercise training could be augmented by PST supplementation. Moreover, the in vitro study showed that PST treatment remarkably promoted slow-twitch fibers formation, angiogenic factor expression, and mitochondrial function in C2C12 myotubes. Collectively, our results suggest that PST promotes skeletal muscle adaptations to exercise training thereby enhancing the endurance capacity.

## 1. Introduction

Regular endurance exercise not only improves endurance capacity but also prevents metabolic diseases such as obesity, type 2 diabetes, metabolic syndrome, and cardiovascular diseases [1,2]. Exercise-induced skeletal muscle adaptions play a key role in enhanced exercise performance and metabolic benefits [3]. Skeletal muscle is composed of myofibers with distinct contractile and metabolic properties, thereby enabling different types of muscle to perform specific motor activities [4,5]. Myofibers are classified into slow-twitch, also known as type I, and fast-twitch, also known as type II fibers, according to their contractile properties and the expression of specific isoforms of myosin heavy chain (MHC). There are four MyHC isoforms including MyHC-I, MyHC-IIa, MyHC-IIx, and MyHC-IIb expressed in skeletal muscle. As such, fibers predominantly expressing MyHC-I are termed type I fibers, while fibers predominantly expressing MyHC-IIa, MyHC-IIx, and MyHC-IIb are termed type IIA, type IIX, and type IIB fibers, respectively. Type I and type IIA fibers display oxidative metabolism and are resistant to fatigue, while type IIX and type IIB fibers exhibit glycolytic metabolism and are quickly fatigable [6]. Skeletal muscle has high plasticity. In humans, there are several internal or external variables including physical activity [7] or inactivity [8], changes in neural stimulus [9], aging [10], and diet or obesity [11,12] that can influence MHC isoform expression, thereby inducing fiber type transition. Regular endurance exercise could remodel skeletal muscle by inducing a substantial switch in composition from fast-twitch, glycolytic fibers to the more oxidative, slow-twitch fibers [13]. In addition to muscle fibers transition, angiogenesis and mitochondrial biogenesis in skeletal muscle are also included in endurance exercise-induced skeletal muscle adaptions, all of them contribute to the exercise-induced improvement of endurance capacity and metabolic status [2,3].

Despite the well-known benefits of regular exercise in physical performance and metabolic status, regular participation in physical exercise is relatively low in the general population [14]. Therefore, exercise enhancers that can augment exercise-induced muscle adaptions, thereby partially substituting for physical exercise, have attracted much attention [15]. In recent years, some dietary polyphenols, such as (-)-epicatechin and apple polyphenols were found to promote exercise capacity through increasing skeletal muscle mitochondrial biogenesis and angiogenesis, and remodeling muscle fiber phenotype [16,17]. Resveratrol (RSV), a naturally occurring polyphenol, was found in a variety of plants, especially in the peel of grapes and peanuts [18]. Some previous studies suggested that RSV supplementation could increase exercise endurance in rodents [19,20,21] and improve muscle fatigue resistance in older adults [22]. RSV supplementation could also promote a fast to slow MyHC isoform shift in MDX mice [23] and evoke an increase in MyHC-I gene expression of mature myotubes [24]. However, the poor water solubility and low bioavailability of RSV limit its practical applications [25,26]. Pterostilbene (PST) is a natural dimethylated analog of RSV, it has greater lipophilicity and a higher in vivo bioavailability than RSV [27]. RSV and PST exhibit many pharmacological similarities including analgesia, antiaging, antidiabetic, anti-inflammation, antioxidation, neuroprotective activities, and so on [28,29]. Moreover, PST was found to be more metabolically stable and exhibited stronger pharmacological activities than that of RSV [30]. Therefore, PST appears superior to RSV in biomedical applications including improving exercise endurance. However, there were no prior reports on the effects of PST supplementation on endurance capacity.

Thus, the purpose of this study was to determine if PST augments endurance training-induced skeletal muscle adaptations, thereby promoting endurance capacity in Sprague Dawley (SD) rats. In addition, the effects of PST on muscle fiber transformation, paracrine regulation of angiogenesis, and mitochondrial function were also investigated in vitro using C2C12 myotubes.

## 2. Results

### 2.1. PST Promotes Exercise Training-Induced Endurance Capacity

**Figure 1:**
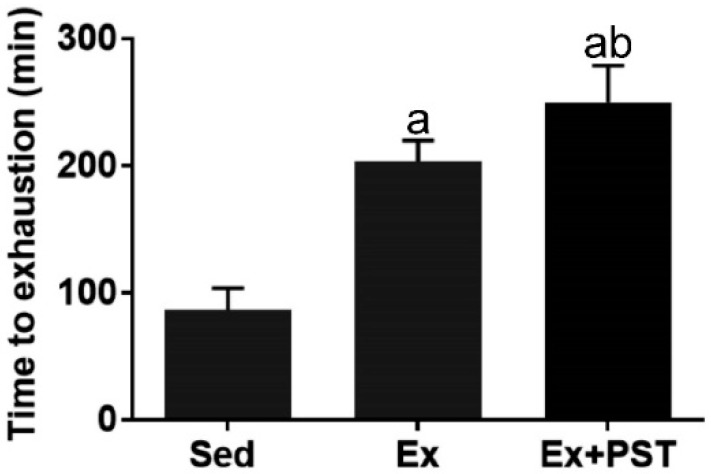
Effect of pterostilbene (PST) supplementation on endurance capacity in exercise training rats. After the run-to-fatigue experiments, time to exhaustion (TTE) was recorded. Data are expressed as mean ± SD, a—*p* < 0.05 compared with Sed group; b—*p* < 0.05 compared with Ex group.

To examine the effect of PST supplementation and exercise training on endurance capacity in rats, we performed an endurance test at the end of the intervention. The exercise endurance was indicated by TTE. As shown in Figure 1, the TTE of the Ex group was significantly longer by 2.21-fold (*p* < 0.05) as compared with the Sed group, indicating that exercise training markedly enhanced endurance capacity. Compared to the Ex group, the TTE of the Ex + PST group was significantly longer by 1.30-fold (*p* < 0.05), suggesting that PST supplementation during exercise training period dramatically augmented the effect of exercise training on endurance capacity.

### 2.2. PST Increases Proportion of Slow-Twitch Fibers in Exercise Training Rats

**Figure 2:**
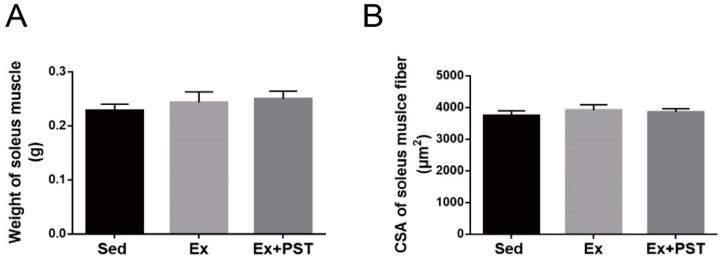

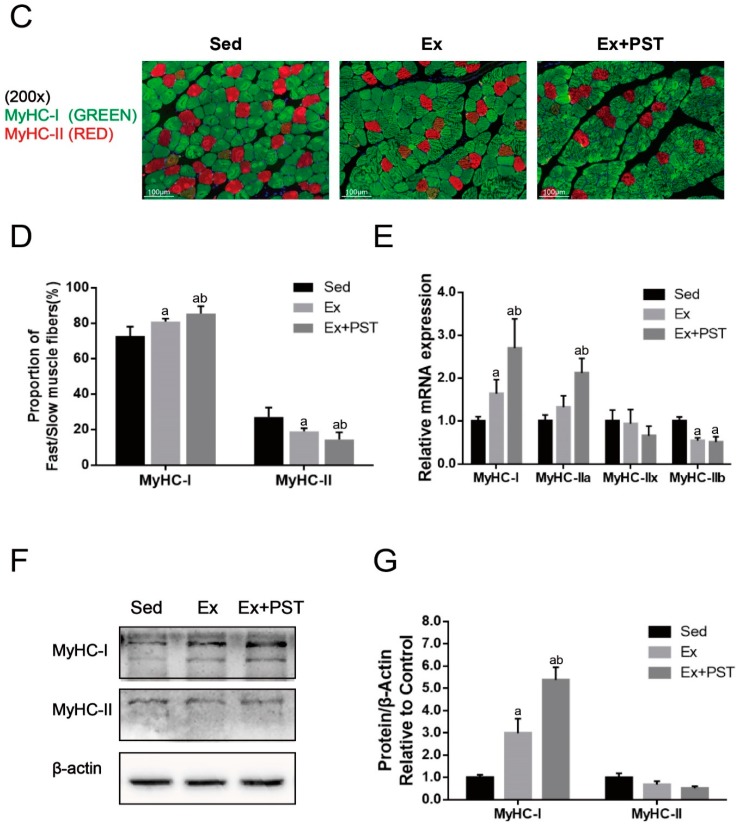
Effects of pterostilbene (PST) supplementation on the compositions of muscle fiber types in the soleus of exercise training rats. Soleus weight to body weight ratio (**A**) and a cross-sectional area (CSA) of soleus was measured (**B**). Changes in muscle fiber types of soleus were shown by immunofluorescent staining (**C**,**D**), real-time quantitative PCR (**E**) and Western blot (**F**,**G**). Scale bar, 100 μm. Data are expressed as mean ± standard deviation (SD), a—*p* < 0.05 compared with the Sed group; b—*p* < 0.05 compared with the Ex group.

In this study, we first evaluated the changes in soleus muscle mass and the cross-sectional area (CSA) of soleus muscle fibers and there were no significant differences between these groups (*p* > 0.05, Figure 2A,B). Then, we analyzed the skeletal muscle fiber composition of the soleus muscle by immunofluorescence staining. The results showed that the proportion of slow-twitch fibers in the rats of the Ex group was higher than that of the Sed group, concomitant with a decrease in the proportion of fast-twitch fibers (*p* < 0.05, Figure 2C,D). Compared to the Ex group, the Ex + PST group has a higher proportion of slow-twitch fibers and a lower proportion of fast-twitch fibers (*p* < 0.05, Figure 2C,D). It demonstrates that exercise training induces fast-to-slow fiber type transition, and this effect could be amplified by PST treatment.

To further confirm the muscle fiber type conversion, we performed quantitative real-time PCR and western blot to quantify the expression of fiber type-specific MyHC isoforms. As shown in Figure 2E, exercise training induced higher expression of MyHC-I and MyHC-IIa mRNA (*p* < 0.05) and a lower expression of MyHC-IIb mRNA (*p* < 0.05) in the soleus muscle as compared with the Sed group, and these changes were further augmented by PST treatment (*p* < 0.05). Moreover, the results of immunoblotting were consistent with the findings of MyHC isoforms mRNA expression (Figure 2F,G). Taken together, these data reveal that PST supplementation enhances exercise training stimulated fast-to-slow fibers transition. 

### 2.3. PST Enhances Muscular Angiogenesis in Exercise Training Rats

**Figure 3:**
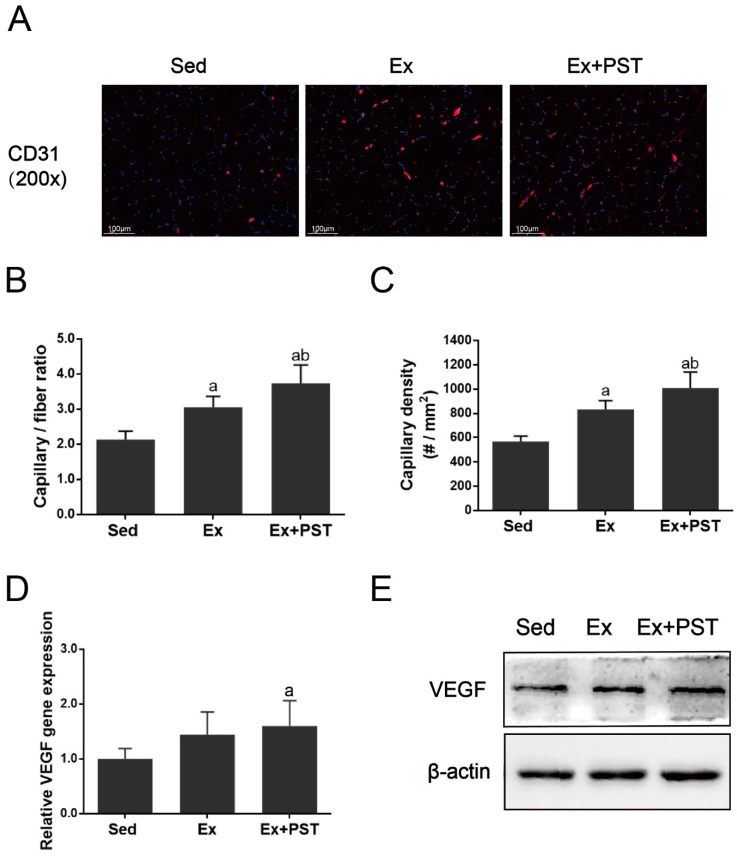

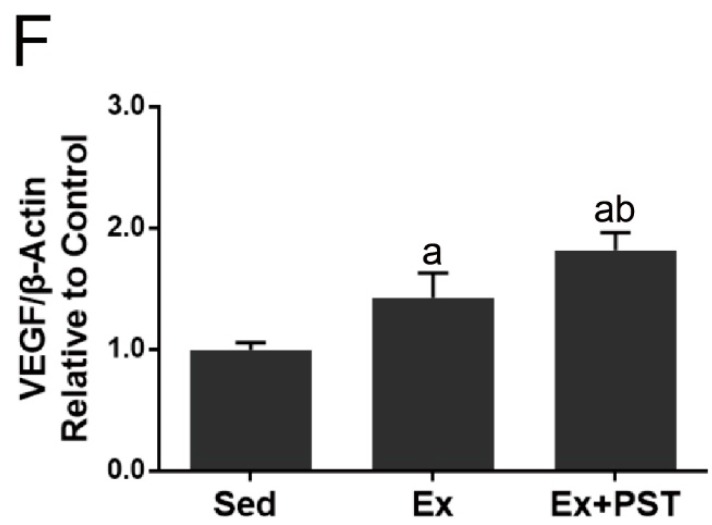
Effects of pterostilbene (PST) supplementation on muscular angiogenesis in exercise training rats. Paraffin sections were obtained from soleus muscles and stained for CD31 (a marker of endothelial cells) (200 × objective) (**A**). Capillary to fiber ratio (**B**) and vascular density (**C**) were determined by counting the CD31+ vascular structures from three independent fields of view per animal. Genes expressions (**D**), representative western blotting bands (**E**) and their quantitative analysis (**F**) of VEGF from soleus. Scale bar, 100 μm. Protein expression levels were normalized to the expression of β-actin. Data are expressed as mean ± SD, a—*p* < 0.05 compared with Sed group; b—*p* < 0.05 compared with Ex group.

In this study, we first performed immunofluorescence staining of CD31 in the soleus muscle to evaluate angiogenesis as indicated by the capillary-to-fiber ratio (CFR) and capillary density (CD). The CFR and CD were expressed as capillaries per muscle fiber and capillaries per unit area (mm^2^) of muscle fiber, respectively. As shown in Figure 3A–C, exercise training resulted in a higher CFR and CD in soleus muscle as compared with the Sed group (*p* < 0.05), and PST supplementation remarkably reinforced the proangiogenic effect of exercise training in skeletal muscle (*p* < 0.05). 

As VEGF is the most important regulator of angiogenesis, we next measured VEGF expression at the mRNA and protein levels in the soleus muscle. The results showed that the expressions of VEGF mRNA and protein in the soleus muscle of the Ex group were higher than that of the Sed group (*p* < 0.05), while the Ex + PST group had a much higher level of VEGF mRNA and protein than that of the Ex group (*p* < 0.05) (Figure 3D–F).

### 2.4. PST Promotes Slow-Twitch Fiber Formation in C2C12 Myotubes

**Figure 4:**
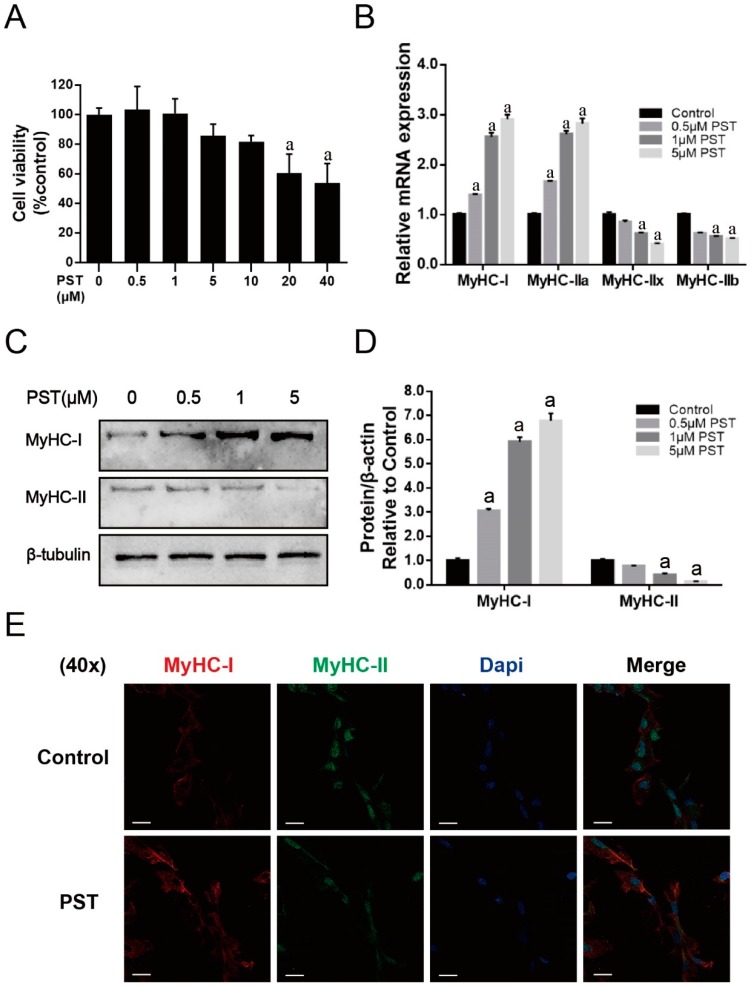
Effects of pterostilbene (PST) treatment on muscle fiber types in C2C12 myotubes. Cell viability (**A**) was measured using a CCK-8 assay. Gene expressions of MyHC-I, MyHC-IIa, MyHC-IIx, and MyHC-IIb (**B**). Representative images of western blots of genes related to muscle fiber type transition (**C**) and their quantitative analysis (**D**) in C2C12 myotubes treated without or with 0.1–5 μM PST. Representative immunofluorescence images of C2C12 myotubes treated without or with 1 μM PST (**E**). The cells were labeled with primary antibody for MyHC-I (red) and MyHC-II (green), as well as with DAPI (blue). Scale bar, 20 μm. Protein expression levels were normalized to the expression of β-tubulin. Data are expressed as mean ± SD, a—*p* < 0.05 compared with the control group.

To verify the observed regulatory effect of PST on skeletal muscle fiber type composition in exercise-trained rats, we further investigated the effect of PST treatment on the expression of fiber type-specific MyHC isoforms in C2C12 myotubes in vitro. PST did not markedly affect the cell viability of C2C12 cells at concentrations below 10 μM (Figure 4A). As shown in Figure 4B, PST treatment significantly increased the expressions of MyHC-I and MyHC-IIa mRNA, and decreased the expressions of MyHC-IIx and MyHC-IIb mRNA (*p* < 0.05). The results of immunoblotting showed that the MyHC-I protein level was significantly increased, while the MyHC-II protein level was markedly decreased after treatment with PST (*p* < 0.05, Figure 4C,D). Furthermore, the effect of PST treatment on the expression of fiber type-specific MyHC isoforms in C2C12 myotubes was further confirmed by immunofluorescence staining (Figure 4E). Collectively, these results indicated that PST treatment could promote slow-twitch fiber formation in C2C12 myotubes.

### 2.5. PST Increases VEGF Production in C2C12 Myotubes

**Figure 5:**
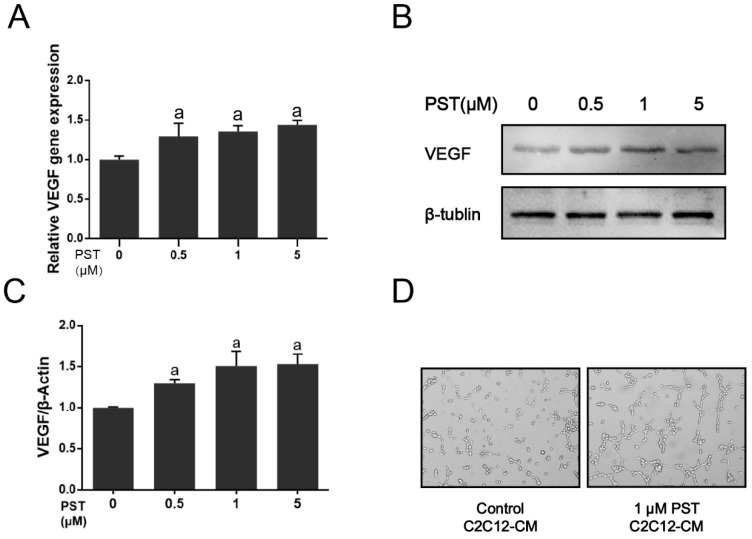
Effects of pterostilbene (PST) treatment on muscular angiogenesis in vitro. Gene expressions (**A**), representative western blotting bands (**B**), and their quantitative analysis (**C**) of VEGF in C2C12 myotubes treated without or with 0.1–5 μM PST. Tube formation assay test of murine endothelial cells (**D**). SVEC4-10 cells were treated with conditioned media (vehicle or 1 μM PST treated C2C12 myotubes) for 3–4 h. Similar results were obtained from 3–6 experiments. Protein expression levels were normalized to the expression of β-tubulin. Data are expressed as mean ± SD, a—*p* < 0.05 compared with the control group.

To clarify the mechanism underlying the effect of PST supplementation on muscular angiogenesis in exercise-training rats, we further investigated the paracrine effect of PST on angiogenesis in C2C12 myotubes. As shown in Figure 5A–C, PST treatment significantly increased mRNA and protein levels of VEGF in C2C12 myotubes as compared with the vehicle-treated control (*p* < 0.05). Moreover, treatment of SVEC4-10 cells with conditioned media from PST-treated C2C12 myotubes stimulated tube formation in 3–4 h (Figure 5D). These results demonstrate that PST treatment could increase angiogenic factor VEGF production in C2C12 myotubes to promote angiogenesis in a paracrine fashion.

### 2.6. PST Increases Muscle mtDNA Copy Numbers Both In Vivo and In Vitro

**Figure 6:**
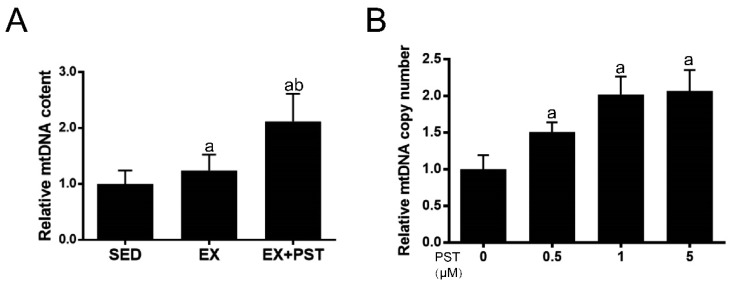
Effects of pterostilbene (PST) treatment on mitochondrial DNA copy number in muscle. Mitochondrial DNA copy number in the soleus (**A**) and C2C12 myotubes (**B**) were measured. Data are expressed as mean ± SD, a—*p* < 0.05 compared with the Sed group; b—*p* < 0.05 compared with the Ex group.

Since slow-switch fibers have higher mitochondrial content as compared with fast-twitch fibers, we measured mitochondrial content as indicated by mtDNA copy numbers in soleus or C2C12 myotubes to confirm that PST treatment promotes slow-switch fiber formation. In the in vivo study, the mtDNA copy numbers in soleus muscle were significantly higher in the Ex + PST group than that in the Ex group (*p* < 0.05, Figure 6A). In the in vitro study, PST treatment markedly increased mtDNA copy numbers in C2C12 myotubes (*p* < 0.05, Figure 6B). These data were in line with the changes in slow-twitch fiber formation triggered by PST treatment.

### 2.7. PST Enhances Mitochondrial Oxidative Metabolism in C2C12 Myotubes

**Figure 7:**
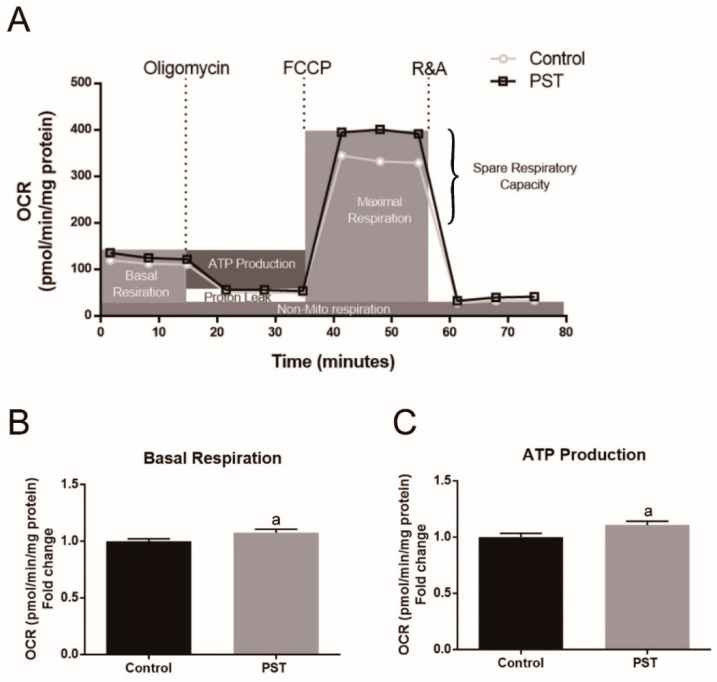

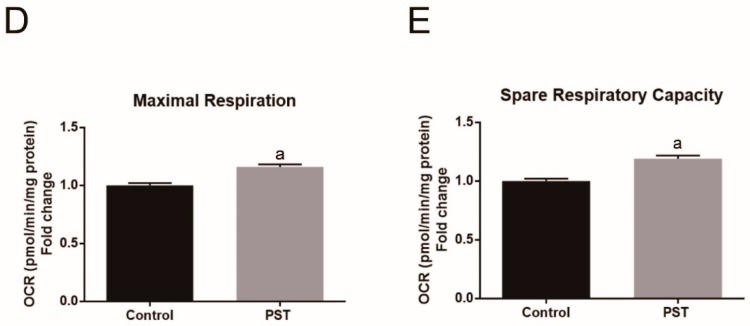
Effects of pterostilbene (PST) treatment on mitochondrial function in C2C12 myotubes. Oxygen consumption rate of C2C12 myotubes treated without or with 1 μM PST (**A**). The data obtained in panel A were used to calculate the basal respiration (**B**), ATP production (**C**), maximal respiration (**D**) and spare respiratory capacity (**E**). Data are expressed as mean ± SD and analyzed via Student’s *t*-test, a—*p* < 0.05.

Slow-twitch fibers have a higher oxidative capacity due to more mitochondria content than fast-twitch fibers. In view of PST promoting slow-twitch fiber formation observed both the in vivo and in vitro study, we further investigated the effect of PST treatment on mitochondrial function in C2C12 myotubes using the Seahorse XFp analyzer. The results showed that PST treatment resulted in a significant increase in basal respiration, ATP production, maximal respiration, and spare respiratory capacity in C2C12 myotubes (*p* < 0.05, Figure 7A–E), indicating the promotion of mitochondrial oxidative metabolism. These data were consistent with the finding of mtDNA copy numbers in C2C12 myotubes after treatment with PST.

## 3. Discussion

Exercise mimetics have attracted considerable attention due to their potential applications in enhancing exercise performance and protecting against metabolic diseases, especially some naturally occurring bioactive ingredients in food [3,15]. RSV and its natural dimethylated analog PST both exhibit many health benefits such as antiinflammation, anticarcinogenesis, antidiabetic, and anticardiovascular diseases effect [28,29]. However, PST may be superior to RSV in practical applications because of its higher oral bioavailability than RSV [27]. In the current study, we showed that endurance exercise resulted in increases in endurance capacity, the proportion of slow-twitch fibers, muscular angiogenesis and mitochondrial biogenesis in rats, and these effects induced by exercise training could be augmented by PST supplementation. Additionally, PST treatment could promote slow-twitch fiber formation, angiogenic factor VEGF expression and mitochondrial biogenesis in C2C12 myotubes. These data indicate that PST is a promising exercise enhancer.

It has been widely demonstrated that endurance exercise can lead to a series of adaptations in skeletal muscle, such as fiber type transition from fast-twitch to slow-twitch, mitochondrial biogenesis, and angiogenesis, thereby improving endurance performance and metabolic homeostasis [2,3,31]. In the current study, we examined the effects of endurance exercise in the form of treadmill running on endurance capacity and skeletal muscle adaptations in rats. The results showed that treadmill running significantly improved endurance capacity as evidenced by the increased TTE. Meanwhile, we found that treadmill running remarkably induced a fast-to-slow shift of muscle fiber type composition in the soleus muscle, as indicated by an increased proportion of slow-twitch fibers with a concomitant reduction in fast-twitch fibers as well as the altered expression of fiber type specification myosin heavy chain isoforms. This result is consistent with the findings of several previous studies [32,33]. Moreover, slow-twitch fibers have higher mitochondrial content and capillarity compared with fast-twitch fibers, thus the changes in muscle fiber type are also reflected in muscular mitochondrial content and angiogenesis. It has been demonstrated that endurance exercise training could lead to skeletal muscle mitochondrial biogenesis both in rodents and humans [34,35,36,37]. In line with these findings, our study found that treadmill running significantly increased the amount of mtDNA copy number in soleus. Several lines of evidence have demonstrated that endurance exercise induces angiogenesis in muscles thereby resulting in an increase in blood flow to muscle to provide additional supply of oxygen and nutrients required by exercise, and that increased VEGF expression from muscle fibers plays a pivotal role in exercise-induced angiogenesis in skeletal muscle through paracrine actions [38,39,40,41,42]. In the present study, we also found that treadmill running induced a notable angiogenic response in the soleus muscle, as evidenced by the increased capillarity and the elevated expression of VEGF. Taken together, our findings further support the notion that muscular adaptions including fiber type transformation, mitochondrial biogenesis, and angiogenesis in response to endurance exercise are the basis for the improvement of endurance performance.

Although it has been widely accepted that regular exercise has great benefits, such as enhanced physical performance and metabolic homeostasis, there are numerous circumstances in which the ability to exercise is limited. Therefore, pharmacologic intervention, which could mimic exercise-induced muscle adaptation, should be considered as a promising strategy. AMPK has been shown to play a pivotal role in exercise-induced skeletal muscle adaptations [43,44]. Moreover, AICAR (5-amino-1-β-d-ribofuranosyl-imidazole-4-carboxamide), a chemical AMPK activator, has been shown to increase endurance capacity in animal studies [45]. However, it should be noted that the application of AICAR is highly debated because of its potential side effects [46]. In recent years, the exercise-mimicking effects of natural products, especially for bioactive ingredients of food, such as RSV, epigallocatechin gallate, and capsaicin which could activate AMPK, have attracted much attention owing to their safety [47]. RSV belongs to a stilbene class of polyphonic compounds, commonly found in grapes, berries, peanuts, and other plant foods. During the past two decades, RSV has been widely studied and associated with many potential health benefits [48,49]. RSV has been shown to promote endurance performance in mice [19,20,21] and exercise-induced cellular and functional adaptions of skeletal muscle in elderly people [22]. However, the poor oral bioavailability of RSV in humans has been a major limitation for clinical application [50]. PST as a natural dimethylated analog of RSV, has several key advantages over RSV for practical applications, including superior biological activity, better oral bioavailability, and slower metabolism in the body [27]. Accordingly, it is valuable to investigate the effects of PST on exercise-induced skeletal muscle adaptions.

In the current study, we found that PST supplementation remarkably enhanced TTE in exercise training rats, and increased the proportion of slow-twitch fibers and capillarization, along with increasing the expression of slow-twitch fiber specific myosin heavy chain isoform MyHC-I and angiogenic factor VEGF, as well as mtDNA copy number in the soleus muscle. Moreover, our in vitro data showed that PST treatment significantly increased the expression of MyHC-I and VEGF, mtDNA copy number, basal respiration, ATP production, maximal respiration, and spare respiratory capacity in C2C12 myotubes. Given that the mtDNA copy number is generally regarded as a surrogate of mitochondrial content, its increase suggests that exercise training and PST enhance skeletal muscle mitochondrial function by promoting mitochondrial biogenesis, while the individual mitochondrial function may not be affected by exercise training and PST treatment. In addition, it was found that conditioned media from PST-treated C2C12 myotubes induced endothelial cells SVEC4-10 to form a tube in culture. These results suggest that PST treatment promoted slow-twitch fiber formation, muscular angiogenesis, and mitochondrial function in C2C12 myotubes. Collectively, our data indicate that PST supplementation could augment exercise training-induced skeletal muscle adaptions and endurance capacity.

In summary, we report the novel finding that PST supplementation notably promoted exercise training-induced endurance capacity in rats. This improvement in endurance capacity is likely to be the result of the pleiotropic effects of PST on skeletal muscle adaptions in response to exercise including muscle fiber type transition, mitochondrial biogenesis, and angiogenesis. In the future, the underlying molecular mechanisms of action of PST and its potential clinical application as an exercise enhancer warrant further investigation.

## 4. Materials and Methods 

### 4.1. Animal Maintenance

All experimental procedures described herein were approved by the Army Medical University Institutional Animal Care and Use Committee (Chongqing, China; Approval SYXC-2017-0002). A total of 18 six-week-old male Sprague Dawley (SD) rats were obtained from the Laboratory Animal Centre of the Army Medical University (Chongqing, China). Animals were housed under standard environmental conditions (20–22 °C, 12 h light/dark cycle) and provided food and water ad libitum. After one week of adaptation, rats were randomly divided into three groups with 6 rats in each group: Sedentary control group (Sed), exercise training group (Ex), and exercise training combined with 50 mg/kg PST treatment group (Ex + PST). PST (HPLC purity ≥98%, A0752, CHENGDU MUST, China) was dissolved in distilled water and was given by oral gavage once a day, while the other groups were treated with the equal volume of distilled water as a vehicle by oral gavage. 

### 4.2. Exercise Training and Exercise Endurance Test

One hour after vehicle or PST administration, rats in the Ex and Ex + PST groups performed regular running on a motorized treadmill at a speed of 20 m/min. The exercise training program was as follows: 20 m/min for 20 min on the first day followed by a 10 min increase per day to a maximum of 60 min/d, training 5 d and rest 2 d per week. 

After 4 weeks of intervention, all rats ran on the motorized treadmill at 20 m/min until they were exhausted. A rat was deemed to be fatigued when it was no longer able to continue to run on the treadmill as judged by the rat spending >20 consecutive seconds on the electrical stimulus and resistant to mechanical prodding. The time from beginning to exhaustion is defined as the time-to-exhaustion (TTE) that is used to indicate exercise endurance. 

Immediately after the run-to-fatigue exercise, rats were euthanized and weighed. The soleus muscle was excised, weighed, immediately frozen in liquid N2, and stored at −80 °C until processing and analysis.

### 4.3. Cell Culture and Treatment

C2C12 myoblasts were cultured in Dulbecco’s Modified Eagle’s Medium (DMEM; Gibco-Invitrogen, Carlsbad, CA, USA) supplemented with 10% fetal bovine serum (FBS; HyClone, Logan, UT, USA) and 1% penicillin–streptomycin (ST488, Beyotime, Shanghai, China) at 37 °C in a saturated humidity atmosphere containing 95% air and 5% CO_2_. When the cells were grown to 80–90% confluence, cell differentiation was induced using a differentiation medium containing DMEM with 2% horse serum (Gibco, Carlsbad, CA, USA). After 1 d of differentiation, the cells were then treated with PST (0.5, 1, or 5 µM) (HPLC purity ≥98%, P1499, Sigma-Aldrich, St Louis, MO, USA) or vehicle (DMSO; Sigma, St Louis, MO, USA). The medium was then replaced with a fresh differentiation medium daily for 5 days before analysis.

### 4.4. Cell Viability Assay

CCK-8 kits were used to measure cell viability following the manufacturer’s protocol. Briefly, C2C12 myoblasts were cultivated in a 96-well plate at a density of 8000 cells/well and treated with PST as described above. At the end of treatment, CCK-8 (10 μL/well) was added to each well of the plate, and the plate was incubated at 37 °C for 2 h in the incubator. Cell viability was detected by absorbance measurements at a wavelength of 450 nm using an Infinite M200 Microplate Reader (Tecan, Mannedorf, Switzerland). 

### 4.5. In Vitro Angiogenesis Assay

The in vitro angiogenesis assay was performed with a slight modification as described previously [51]. Briefly, conditioned media were collected from PST or vehicle-treated C2C12 myotubes. Mouse endothelial <4–10 cells at a concentration of 4 × 10^5^ cells/mL were plated in matrigel-coated 12-well plates (500 μL/well), and then were treated for 3–4 h with C2C12 myotubes conditioned media (250 μL/well), followed by observation of tube formation using an Olympus inverted microscope.

### 4.6. RNA Extraction, Reverse Transcription, and Real-Time PCR

Total RNA was isolated from soleus muscle or C2C12 myotubes using the Trizol reagent (Invitrogen, Carlsbad, CA, USA) according to the manufacturer’s instructions and quantified using the NanoDrop 2000 spectrophotometer (Thermo Fisher Scientific, Carlsbad, CA, USA). For real-time PCR, RNA was retrotranscribed using PrimeScript RT Master Mix (Takara, Kusatsu, Japan) according to the manufacturer’s directions. Gene-specific primers were designed by Sangon Biotech (Shanghai, China) Co., Ltd. Quantitative real-time PCR was performed with SYBR Green PCR Master Mix (Applied Biosystems, Carlsbad, CA, USA), according to the manufacturer’s instructions. The expression level of the target gene was normalized to the level of β-actin. The gene-specific premier sequences are listed in Table 1.

### 4.7. Determination of Mitochondrial DNA Copy Number 

The determination of mitochondrial DNA copy number was performed as described previously [52]. Briefly, mtDNA was extracted from soleus muscles and C2C12 myotubes using the Mito DNA Extraction Kit (Genmed Scientifics Inc., Wilmington, MD, USA) according to the manufacturer’s instructions. Primers specific for the coding region of mtDNA (cytochrome c oxidase II (COX2)) were used for the quantification of the mtDNA copy number, whereas primers specific for the 18S nuclear gene were used for standardization. Relative transcript abundance was calculated using the delta–delta cycle threshold (ΔΔCt) method.

### 4.8. Western Blot

Soleus muscle and C2C12 myotubes protein extraction were performed using Ripa Buffer and Protease/Phosphatase Inhibitor Cocktail (Roche Applied Science, Indianapolis, IA, USA) and protein quantification using the Bradford method. Protein samples were then subjected to 10–15% SDS-PAGE, transferred to PVDF membranes (Bio-Rad, Hercules, CA, USA), blocked with 5% skim milk at room temperature for 2 h, then incubated overnight at 4 °C with primary antibodies of Slow Skeletal Myosin Heavy chain/MyHC-I (1:1000, ab11083, Abcam, Cambridge, UK), Fast Skeletal Myosin Heavy chain/MyHC-II (1:1000, ab91506, Abcam, Cambridge, UK), and Vascular Endothelial Growth Factor (1:1000, ab1316, Abcam, Cambridge, UK). Membranes were washed in TBST three times and incubated with relative secondary antibodies (Peroxidase-Conjugated Goat anti-Rabbit IgG; Peroxidase-Conjugated Goat anti-Mouse IgG, ZSGB-Bio, Wuhan, China) for 1 h at room temperature. Subsequently, the membranes were washed in TBST three times and protein bands were visualized with enhanced chemiluminescent agents using Fusion FX (Vilber Lourmat, Marne La Vallée, France) and quantified with Gel-Pro Analyzer (Media Cybernetics, Rockville, USA). β-actin (1:1000, sc-47778, SantaCruz, Dallas, TX, USA) or β-tubulin (1:1000, 2128s, Cell Signaling, Beverly, MA, USA) were used as loading controls. 

### 4.9. Immunofluorescence Staining

Immunohistochemistry techniques were used for fiber type determination and capillary density analysis. Soleus muscle tissues fixed with 10% formaldehyde were dehydrated, xylene-mounted, paraffin-embedded, and cross-sectioned (5 μm) for histological immunostaining. Briefly, sections were blocked with 10% donkey serum (30 min, room temperature). Afterward, sections were incubated overnight at 4 °C with primary antibodies: MyHC-I (1:500, ab11083, Abcam, Cambridge, UK), MyHC-II (1:100, ab91506, Abcam, Cambridge, UK), and CD31 (1:200, GB12063, Servicebio, Wuhan, China). Following washing with PBS, slides were incubated with relative secondary antibodies (Alexa Fluor 488 goat-anti mouse IgG, Alexa Fluor CY3 Goat Anti-Rabbit IgG, Servicebio, Wuhan, China). The sections were analyzed by Multi-Photo Laser Scanning Microscopy (Zeiss LSM780NLO) for calculating cross-sectional area, MyHC-I and MyHC-II fiber percentages. Similarly, the capillary density and capillary-to-fiber counts were averaged from three independent fields of view per animal at ×200 magnification, and ~115 fibers/image were counted for each field of view.

PST or vehicle-treated C2C12 myotubes were obtained as described before. For indirect immunofluorescence, C2C12 myotubes were fixed in 4% paraformaldehyde, permeabilized with 0.25% Triton X-100, and blocked with 1% BSA/PBST for 1 h at room temperature, and incubated overnight in 4 °C with primary antibodies MyHC-I (1:100, ab11083, Abcam, Cambridge, UK) and MyHC-II (1:100, ab91506, Abcam, Cambridge, UK). Relative secondary antibodies (Alexa Fluor 488-labeled Goat Anti-Rabbit IgG, Cy3-labeled Goat Anti-Mouse IgG Beyotime, Shanghai, China) were used in 1:500 at room temperature for 2 h. The cell nuclei were stained with 4′,6-diamidino-2-phenylindole (DAPI) (Beyotime, Shanghai, China). Fluorescent images were detected using a confocal microscope equipped with a 40× oil immersion objective.

### 4.10. Mitochondrial Function Assay Using the Seahorse XFp Analyzer

Mitochondrial function assay was performed using XFp Analyzer (Seahorse Bioscience, Agilent Technologies, Santa Clara, CA, USA). Briefly, C2C12 myoblasts were plated at a density of 14,000/well in an XFp 8-well plate. After differentiation and treatment with PST as mentioned before, C2C12 myotubes were cultured in a 37 °C non-CO_2_ incubator for 1 h with assay medium, which contained 10 mM glucose, 1 mM pyruvate, and 2 mM glutamine, and were adjusted to pH 7.4 at 37 °C. Components of the Cell Mito Stress Test (Agilent) were used to evaluate the mitochondrial function at the following final concentrations: 1.0 μM oligomycin, 3.0 μM FCCP, and 0.5/0.5 μM rotenone/antimycin A. The oxygen consumption rate (OCR) was measured according to the manufacturer’s instructions. After analysis, cellular protein levels in each well were measured by the Bradford method, and this value was used to normalize OCR.

### 4.11. Statistical Analysis

Data are represented as mean ± SD. Statistical analyses were conducted by one-way ANOVA, followed by Tukey’s test using GraphPad prism 6.0 (GraphPad Software, Inc., La Jolla, CA, USA). *p*-values less than 0.05 were considered to be statistically significant. All experiments were repeated a minimum of three times.

## 5. Conclusions

The findings from this study demonstrate that pterostilbene supplementation significantly promotes exercise-induced skeletal muscle adaptions including fast-to-slow fiber type transition, mitochondrial biogenesis and angiogenesis, thereby enhancing endurance capacity in exercise training rats.

## Figures and Tables

**Table 1:** Primer sets for RT-PCR.

Gene	Forward Primer (5′-3′), Reverse Primer (5′-3′)	Gene	Forward Primer (5′-3′), Reverse Primer (5′-3′)
R-MyHC-I	CAGTCATGGCGGATCGAGAG CGGATTCTCCGGTGATGAGG	M-MyHC-I	GCCTGGGCTTACCTCTCTATCAC CTTCTCAGACTTCCGCAGGAA
R-MyHC-IIa	GCGACAGACACCTCCTTCAAGAAC GTCCAGCCAGCCAGTGATGTTG	M-MyHC-IIa	AAGTGACTGTGAAAACAGAAGCA GCAGCCATTTGTAAGGGTTGAC
R-MyHC-IIx	GCGACAGACACCTCCTTCAAGAAC CCAGCCAGCCAGCGATGTTG	M-MyHC-IIx	GCGAATCGAGGCTCAGAACAA GTAGTTCCGCCTTCGGTCTTG
R-MyHC-IIb	CCATCACTGACGCCGCCATG GTTCTTCTTCATCCGCTCCAGGTG	M-MyHC-IIb	CTTGGTGGACAAACTACAGACT TGCAGAATTTATTTCCGTGAT
R-VEGFA	CAAGGCAGACTATTCAACGG GGCACGATTTAAGAGGGGAA	M-VEGFA	ACCCTGGCTTTACTGCTGTACCT TCATGGGACTTCTGCTCTCCTT
R-β-actin	CCACCATGTACCCAGGCATT CGGACTCATCGTACTCCTGC	M-β-actin	TGGAATCCTGTGGCATCCATGAAAC TAAAACGCAGCTCAGTAACAGTCCG
R-COX2	TGAGCCATCCCTTCACTAGG GTTCATCCTGTTCCTGCTCC	M-COX2	TTTTCAGGCTTCACCCTAGATGA GAAGAATGTTATGTTATGTTTACTCCTA
R-18s rRNA	CACGGGTGACGGGGAATCAG CGGGTCGGGAGTGGGTAATTTG	M-18s rRNA	TAGAGGGACAAGTGGCGTTC CGCTGAGCCAGTCAGTGT
